# Geographical Inequalities and Social and Environmental Risk Factors for Under-Five Mortality in Ghana in 2000 and 2010: Bayesian Spatial Analysis of Census Data

**DOI:** 10.1371/journal.pmed.1002038

**Published:** 2016-06-21

**Authors:** Raphael E. Arku, James E. Bennett, Marcia C. Castro, Kofi Agyeman-Duah, Samilia E. Mintah, James H. Ware, Philomena Nyarko, John D. Spengler, Samuel Agyei-Mensah, Majid Ezzati

**Affiliations:** 1 Department of Environmental Health, Harvard School of Public Health, Boston, Massachusetts, United States of America; 2 MRC-PHE Centre for Environment and Health, Imperial College London, London, United Kingdom; 3 Department of Epidemiology and Biostatistics, School of Public Health, Imperial College London, London, United Kingdom; 4 Department of Global Health and Population, Harvard School of Public Health, Boston, Massachusetts, United States of America; 5 Ghana Statistical Service, Accra, Ghana; 6 Department of Biostatistics, Harvard School of Public Health, Boston, Massachusetts, United States of America; 7 Department of Geography and Resource Development, University of Ghana, Legon, Ghana; 8 Wellcome Trust Centre for Global Health Research, Imperial College London, London, United Kingdom; University of Otago, Wellington, NEW ZEALAND

## Abstract

**Background:**

Under-five mortality is declining in Ghana and many other countries. Very few studies have measured under-five mortality—and its social and environmental risk factors—at fine spatial resolutions, which is relevant for policy purposes. Our aim was to estimate under-five mortality and its social and environmental risk factors at the district level in Ghana.

**Methods and Findings:**

We used 10% random samples of Ghana’s 2000 and 2010 National Population and Housing Censuses. We applied indirect demographic methods and a Bayesian spatial model to the information on total number of children ever born and children surviving to estimate under-five mortality (probability of dying by 5 y of age, 5q0) for each of Ghana’s 110 districts. We also used the census data to estimate the distributions of households or persons in each district in terms of fuel used for cooking, sanitation facility, drinking water source, and parental education. Median district 5q0 declined from 99 deaths per 1,000 live births in 2000 to 70 in 2010. The decline ranged from <5% in some northern districts, where 5q0 had been higher in 2000, to >40% in southern districts, where it had been lower in 2000, exacerbating existing inequalities. Primary education increased in men and women, and more households had access to improved water and sanitation and cleaner cooking fuels. Higher use of liquefied petroleum gas for cooking was associated with lower 5q0 in multivariate analysis.

**Conclusions:**

Under-five mortality has declined in all of Ghana’s districts, but the cross-district inequality in mortality has increased. There is a need for additional data, including on healthcare, and additional environmental and socioeconomic measurements, to understand the reasons for the variations in mortality levels and trends.

## Abstract 2

In a census-based study, Majid Ezzati and colleagues use demographic modeling to estimate district-level variation in under-five mortality across Ghana.

## Author Summary

**Why Was This Study Done?:**

- Deaths among children younger than five years old have been declining worldwide for the past few decades. Children’s death rates in sub-Saharan Africa, although also declining, remain higher than in other regions.
- Ghana has performed better than most other sub-Saharan African countries in terms of reducing child deaths, but we lack information on how much child mortality varies in different parts of Ghana.
- Also, it is not known how factors such as low education of mothers or not having clean water or improved toilet facilities that increase the risk of illness and death among children vary in different parts of Ghana.
- The purpose of our study was to provide information on changes in the child death rate, and the social and environmental factors associated with it, for all of Ghana’s 110 districts between the years 2000 and 2010.

**What Did the Researchers Do and Find?:**

- Information from two national censuses in 2000 and 2010 and sophisticated statistical models were used to make estimates of the child death rate in each district for the years 2000 and 2010 and for change between these two years.
- The risk of children dying before they reach age five was higher in northern Ghana than it was in the southern part of the country.
- Child death rates went down in every district in Ghana between 2000 and 2010, but progress was slower in northern Ghana than in the southern part of the country.
- More women and men in the country had completed primary school education in 2010 than in 2000, and more homes had access to clean water and improved toilet facilities and cleaner cooking fuels like liquefied petroleum gas.
- Use of liquefied petroleum gas for cooking may reduce the risk of child death in Ghana.

**What Do These Findings Mean?:**

- There should be strategies to help districts where young children are at a relatively higher risk of dying, largely those in northern Ghana, to catch up with those districts with lower child mortality.
- Some of the actions needed may be related to social and environmental factors like education, clean sanitation, and cooking fuels. But it is likely that there is an important role for healthcare, which was not investigated in this study.
- It is important for Ghana and other sub-Saharan African countries to improve the measurement and monitoring of child mortality and its social, environmental, and healthcare risk factors at the community level.

## Introduction

Mortality in children under 5 y of age has declined in most countries, with the decline accelerating since 2000 [1,2]. The pace of under-five mortality decline has been slower in sub-Saharan Africa (SSA) than in other regions, and SSA accounts for nearly one-half of global under-five deaths [1,3]. Poverty-related risk factors, including unimproved water and sanitation and household air pollution from solid fuels, are estimated to account for a large proportion of worldwide under-five deaths [4]. Parental, and especially maternal, education is also an important predictor of child survival [5]. These factors may also be associated with within-country disparities in under-five mortality [6,7].

Above and beyond global inequalities, there are important subnational inequalities in under-five mortality in relation to socioeconomic status and geography [6,8–14]. Although studies from health and demographic surveillance systems have reported child mortality at specific sites in some countries [15–18], very few studies have examined under-five mortality at fine spatial resolutions for entire countries, which is relevant for assessing community determinants and interventions [9,14,19,20]. Further, little is known about whether and how subnational variations and trends in under-five mortality are associated with social and environmental factors that have been found to be associated with child survival in individual-level studies [6,7].

Ghana has had one of the largest declines in under-five mortality in SSA [1,3]. Under-five mortality in Ghana dropped from 128 deaths per 1,000 live births in 1990 to 62 in 2015, a 52% reduction [1]. Ghana has also had one of SSA’s best economic performances, with its per-capita gross domestic product growing substantially between 2000 and 2013 [21]. Economic inequality, as measured by the Gini coefficient, increased slightly between 1998 and 2006 [21]. It is unclear whether the national improvement in child survival is benefiting all local communities, how it might be affecting within-country inequalities, and whether it is associated with socioeconomic and environmental improvements or whether it is driven by other factors.

In the analyses presented in this paper, we used geocoded data from two national censuses to estimate under-five mortality in Ghana at the district level in 2000 and 2010, and assessed variations and inequalities in under-five mortality in these two years and changes over the decade. We also analyzed the distributions of social and environmental risk factors of under-five mortality, and their associations with under-five mortality and its change. To our knowledge, this report is one of only a few high-resolution “small-area” studies of under-five mortality and its social and environmental risk factors, and the only one to assess change in small-area units over a period of a decade for an entire country. Small-area analysis helps reveal geographical inequalities in mortality and allows the benchmarking of outcomes in each district against the others.

## Methods

### Data Sources

We used 10% random samples of Ghana’s 2000 and 2010 National Population and Housing Censuses. Using a set of predefined questions, both censuses gathered information on a number of individual- and household-level variables related to socioeconomic factors (e.g., literacy and educational attainment for persons 11 y or older), living environment (e.g., household’s main water supply source, sanitation facility type, and cooking fuel type, as a commonly used metric for household air pollution [4,22]), and children's births and deaths for females 12 y or older. Each record in the census data had information on the census enumeration area (EA), the smallest geographical unit, with an average population of 750. The 2000 and 2010 censuses had nearly 26,000 and 38,000 EAs, respectively. Our analysis was conducted at the district level, the country’s second smallest level of subnational administrative divisions, with EAs mapped to the district of residence. We used districts as units of analysis because they are administrative units for resource allocation and for program implementation. Further, EAs were defined separately in each census and could not be mapped from one census to the other. There were 110 and 170 districts in the 2000 and 2010 censuses, respectively. We merged the 2010 districts, linking them to their original districts that had split since 2000, to create 110 common districts for our analyses. The 110 districts are administratively assembled into ten regions: Ashanti, Brong-Ahafo, Greater Accra, Central, Eastern, Northern, Western, Upper East, Upper West, and Volta.

We used the data to calculate the distribution of households or persons in each district for the following variables that are associated with child survival: household’s main source of cooking fuel (wood, charcoal, other biomass, kerosene, liquefied petroleum gas [LPG], electricity), type of sanitation (toilet) facility usually used by households (improved, unimproved), household’s main source of drinking water (improved, unimproved), maternal education (highest educational grade completed: none, primary, secondary or higher), paternal education (highest educational grade completed: none, primary, secondary or higher), and urban versus rural place of residence. We classified census responses on drinking water source and sanitation facility as improved versus unimproved based on WHO/UNICEF joint monitoring program categories for water supply and sanitation (http://www.wssinfo.org).

### Statistical Methods

#### District-level under-five mortality

Our measure of under-five mortality was the probability of dying by 5 y of age (5q0). The censuses asked all women of childbearing age to report on the total number of children ever born and children surviving. This information was the basis for the application of indirect demographic models to estimate under-five mortality, an approach commonly used by researchers and by national and international statistical and health agencies. The method converts the proportion of deaths among children ever born to women in each 5-y age group of the reproductive period into estimates of the probability of dying by exact ages of childhood, and calculates the number of years before the survey date to which the estimates refer [23,24]. Estimates derived from the two youngest age groups of women (15–19 and 20–24 y) tend to be overestimated compared to the population average because children of women in these age groups tend to have a higher risk of dying than children of older women [23]. Therefore, and following other analyses (including those by the UN Inter-agency Group for Child Mortality Estimation [IGME]) [23], we excluded 5q0 estimates based on reports of women aged 15–24 y. The remaining five estimates (one for each 5-y age group between 25 and 49 y), each with a reference date in years prior to the survey date, were used in our analysis. The reference dates for 5q0 estimates for the 2000 census covered the period 1987–1996; for the 2010 census, the period was 1997–2007.

To obtain a single 5q0 estimate for each district for the index years 2000 and 2010, we fitted a Bayesian space-time model to the five estimates per district from each census. The model included a linear time trend for estimates from each census in each district. The district intercepts and slopes were modeled using the Besag, York, and Mollié (BYM) model [25]. In the BYM model, information is shared both locally (amongst neighboring districts), through spatially structured random effects with a conditional autoregressive (CAR) prior, and globally, through spatially unstructured Gaussian random effects. District-specific intercept and slope values are estimated by the sum of their respective spatially structured and spatially unstructured random effects. The prior distributions in the Bayesian framework allow district-specific parameters to be estimated on the basis of a district’s own data and those of its neighbors. This approach balances between overly unstable within-district estimates and overly simplified aggregate national estimates. Samples from the posterior distributions of the intercepts and slopes were used to estimate 5q0 for the years 2000 and 2010.

#### Correcting 5q0 estimates from summary birth histories

The national 5q0 estimates based on the Ghana censuses alone were different from the UN IGME estimates, which use a larger number of data sources [1], especially for 2000 (the additional sources used in the UN IGME estimates are not available at the district level). We adjusted our estimates to match the UN IGME estimates in 2000 and 2010, because the additional data sources likely help provide more reliable estimates. As detailed above, the linear trends fitted to the census-based national estimates were used to obtain 5q0 in 2000 and 2010. We then applied a correction factor, calculated as the ratio of the national estimate of 5q0 from the UN IGME to that from the census alone, to the 5q0 estimates for each district. The means of the corrected district 5q0 estimates for the 2000 and 2010 censuses were 104.7 and 77.5, respectively, compared to 102.1 and 75.1 deaths per 1,000 live births as estimated by the UN IGME.

#### Statistical analysis

We estimated the associations of under-five mortality with its social and environmental determinants at the district level. We analyzed the associations in 2000 and 2010, as well as the associations of the change in under-five mortality between 2000 and 2010 with changes in these factors. The model for associations in each census year was

Ln(5q0)=α+βX+U+V

where 5q0 is the district-level under-five mortality (per 1,000 live births); *X* is a vector of district-level risk factors (each as percent of households or persons), including cooking fuel type (wood, charcoal, other biomass, kerosene, LPG, electricity), sanitation facility (improved, unimproved), drinking water source (improved, unimproved), maternal education (none, primary, secondary or higher), paternal education (none, primary, secondary or higher), and place of residence (urban, rural); *U* is a district-specific spatially structured random effect; *V* is a district-specific unstructured random effect; and α and β are regression coefficients. When analyzing change in under-five mortality, Ln(5q0) and *X* were replaced with their 2000–2010 differences.

Noninformative normal priors with mean 0 and variance 10,000 were placed on all fixed effects parameters; gamma priors, Gamma(0.5,0.0005), were specified for the precision parameters of all random effects. For each analysis, two chains were run, and convergence was monitored using Brooks-Gelman-Rubin diagnostics [26] and visual inspection of the chains. Following convergence, which was before 10,000 iterations in all analyses, a further 200,000 iterations were run, with thinning to every tenth iteration, yielding final samples of 20,000 iterations for inference.

All analyses were implemented using R2WinBUGS and sqldf libraries in the open-source statistical package R version 3.1.0 (R Project for Statistical Computing) and WinBUGS version 1.4 [27].

## Results

**Fig 1:**
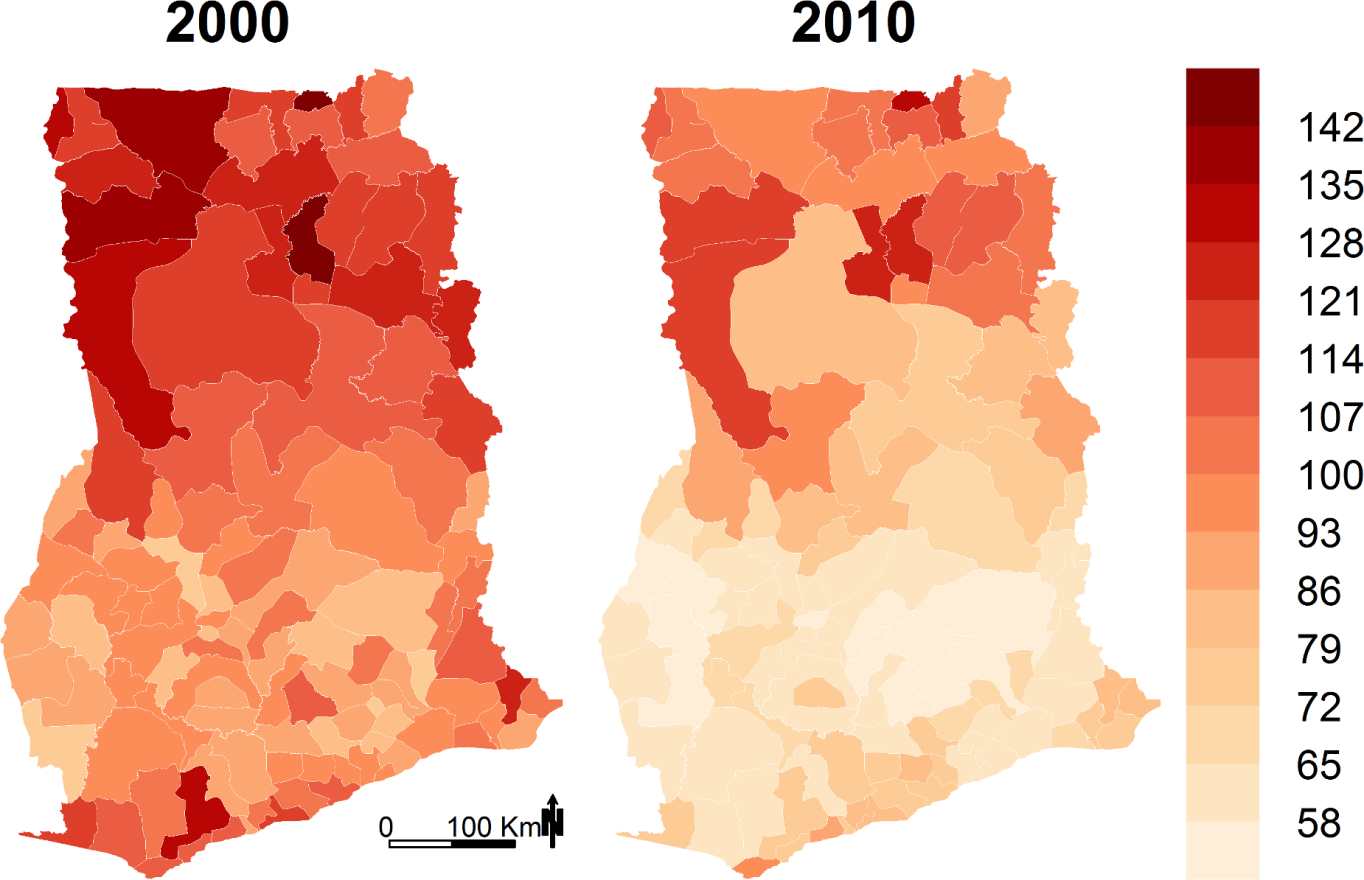
Under-five mortality (deaths per 1,000 live births) by district in 2000 and 2010.

**Fig 2:**
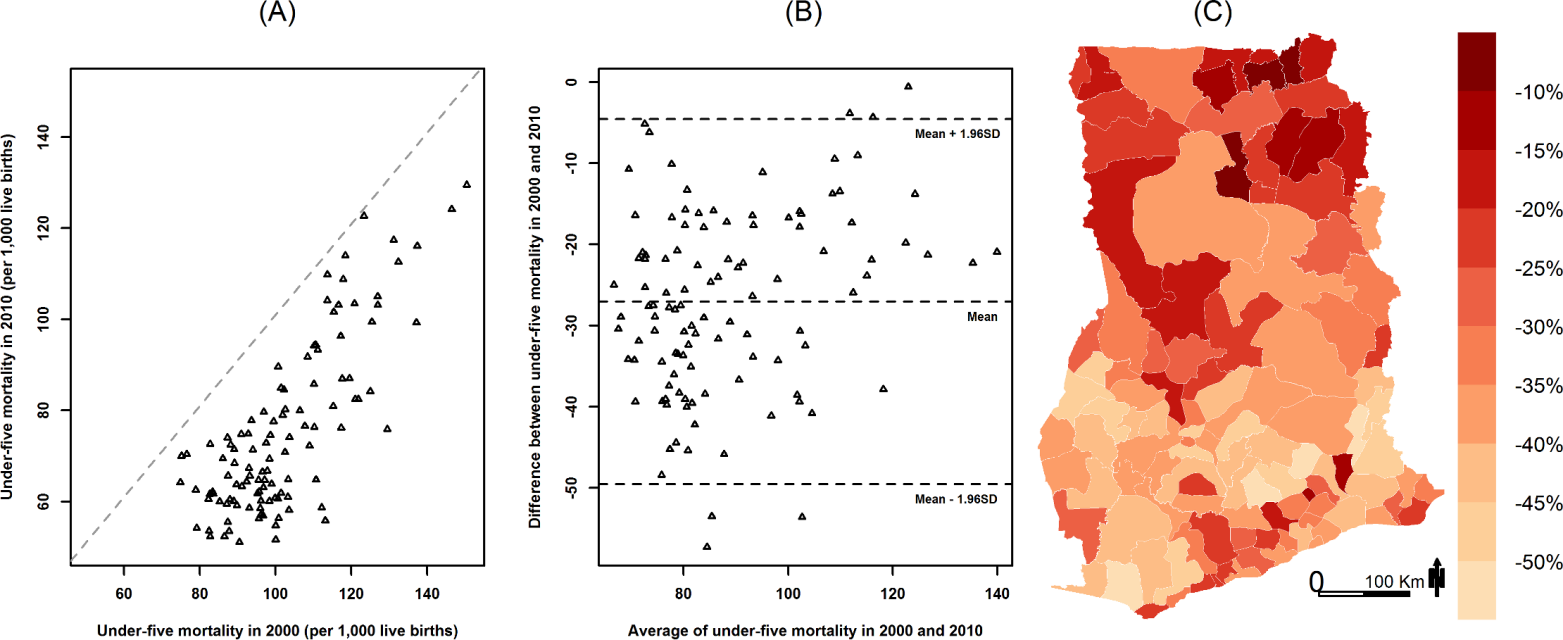
Change in under-five mortality (deaths per 1,000 live births) by district from 2000 to 2010. (A) Under-five mortality (deaths per 1,000 live births) in 2000 versus 2010. (B) Tukey mean-difference plot of under-five mortality (deaths per 1,000 live births) in 2000 and 2010. (C) Percent change in under-five mortality by district from 2000 to 2010.

District under-five mortality estimates and social and environmental factors that may be associated with child survival for the index years 2000 and 2010 are summarized in Table 1. Median district under-five mortality declined from 99 deaths per 1,000 live births in 2000 to about 70 in 2010. In 2000, under-five mortality varied substantially among districts, ranging from about 75 to nearly 150 deaths per 1,000 live births (Fig 1). Under-five mortality was above 100 per 1,000 live births in over half of the districts in 2000. The majority of these districts were in the three northernmost regions of the country (Upper West, Upper East, and Northern). There was also high under-five mortality in areas along the coastline of the Western and Central regions. Under-five mortality declined in all districts between 2000 and 2010 (Fig 2), and was above 100 per 1,000 live births in only 13% of districts in 2010 (Fig 2A). In 2010, under-five mortality was below 70 deaths per 1,000 live births for nearly half of the districts, whereas no district had had under-five mortality this low in 2000 (Fig 1).

**Table 1:** Summary statistics of district population and under-five mortality and its social and environmental risk factors.

Variable	Census 2000	Census 2010	Change from 2000 to 2010
**Population size** (× 10)	13,384 (9,798, 17,251)	16,156 (11,976, 23,163)	2,815 (1,534, 5,301)
**Female (percent of population)**	50.6 (49.8, 51.9)	51.1 (50.4, 52.0)	0.4 (−0.2, 1.0)
**Women of childbearing age (percent of population)**	22.6 (21.8, 23.8)	24.4 (23.5, 25.6)	1.9 (1.2, 2.5)
**Under-five mortality (per 1,000 live births)**	99.2 (90.6, 111.9)	70.2 (60.8, 84.5)	−26.9 (−34.4, −17.9)
**Cooking fuel (percent of households)**			
Wood	77.3 (63.3, 88.0)	61.7 (44.8, 73.4)	−13.8 (−18.8, −8.8)
Charcoal	15.6 (7.3, 26.0)	25.4 (15.6, 38.1)	7.6 (4.5, 12.0)
Other biomass	3.3 (1.6, 4.9)	5.2 (3.4, 6.6)	1.7 (1.0, 2.6)
Kerosene	1.4 (1.1, 1.7)	0.3 (0.2, 0.5)	−1.1 (−1.3, −1.0)
LPG	1.2 (0.5, 2.3)	6.7 (3.4, 11.6)	5.5 (2.9, 8.7)
Electricity	0.4 (0.2, 0.6)	0.3 (0.2, 0.3)	−0.2 (−0.4, 0.0)
**Sanitation facility (percent of households)**			
Improved	42.2 (23.2, 56.4)	83.0 (52.2, 90.7)	30.4 (17.0, 42.8)
Unimproved	57.8 (43.6, 76.8)	17.0 (9.3, 47.8)	−30.4 (−42.8, −17.0)
**Drinking water source (percent of households)**			
Improved	53.2 (40.4, 66.3)	76.3 (66.4, 86.8)	22.6 (13.0, 30.5)
Unimproved	46.8 (33.7, 59.7)	23.7 (13.2, 33.6)	−22.6 (−30.5, −13.0)
**Maternal education (percent of women aged 15–49 years)**			
None	51.7 (42.4, 68.9)	31.2 (22.8, 48.6)	−18.6 (−21.7, −15.5)
Primary	37.1 (19.9, 43.4)	54.4 (36.1, 60.6)	16.9 (13.5, 20.1)
Secondary or higher	11.6 (8.4, 14.0)	12.4 (9.6, 16.6)	1.6 (−0.1, 3.2)
**Paternal education (percent of men aged 15–49 years)**			
None	31.9 (25.3, 55.6)	16.6 (10.9, 37.2)	−14.4 (−17.7, −11.8)
Primary	44.6 (27.9, 51.4)	57.1 (42.9, 63.3)	13.0 (9.8, 16.0)
Secondary or higher	20.2 (16.0, 23.8)	21.7 (17.5, 25.8)	1.9 (−0.3, 3.8)
**Place of residence (percent of households)**			
Rural	74.1 (61.3, 82.6)	66.4 (56.5, 76.8)	−5.2 (−8.9, −0.9)
Urban	25.9 (17.4, 38.7)	33.6 (23.2, 43.5)	5.2 (0.9, 8.9)

Data are presented as median (interquartile range).

From 2000 to 2010, under-five mortality declined by over 40% in some southern districts, but the decline was less in the north, with nearly a third of districts having less than 20% reduction (Fig 2). Districts with the highest mortality in 2000 generally had a smaller decline, exacerbating existing cross-district inequalities, especially for relative inequalities. For example, the difference and the ratio of the highest and lowest percentile of district under-five mortality increased from 76 and 2.0 in 2000 to 78 and 2.5 in 2010, respectively.

**Fig 3:**
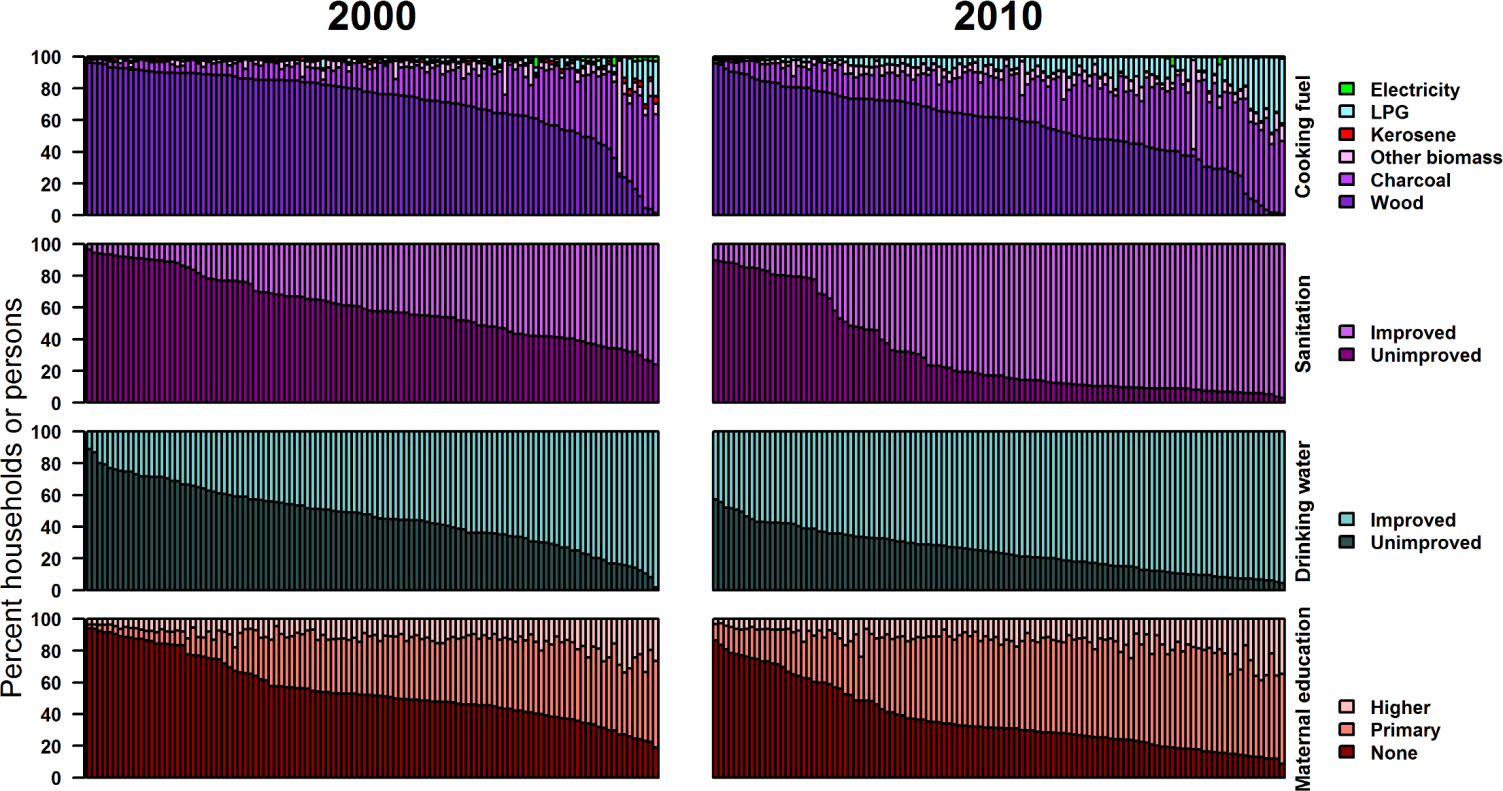
Distributions of household cooking fuel, sanitation facility, drinking water source, and maternal education by district in 2000 and 2010 (percent of households or persons). Each bar represents one district, with districts ordered by decreasing prevalence of the worst category for each census.

From 2000 to 2010, the proportion of households without improved sanitation and drinking water decreased in all districts (Table 1). The share of households with improved drinking water was over 80% in 47 districts, and the share of households with improved sanitation was over 80% in 64 districts, in 2010; virtually no district had had this level of improved drinking water and improved sanitation in 2000 (Fig 3). Despite the improvements, the share of households with improved sanitation was less than 80% in all districts in the Northern, Upper East, and Upper West regions in 2010. There was also some reduction in the use of wood for cooking, largely replaced by charcoal, which emits less health-damaging particulate matter [22,28,29]. The proportion of households using LPG also increased from 6% in 2000 to 18% in 2010. In some districts in the Greater Accra region, over a quarter of households used LPG as the primary cooking fuel in 2010.

More women completed primary education and fewer were illiterate in every district in 2010 compared to 2000 (Fig 3). In over one-half of districts, including in all 24 districts in the northernmost regions, at least one-half of women had not attended school or had not completed primary education in 2000; by 2010, this was the case in 25% of districts. Despite improvements in basic literacy and primary education, the proportion of women who attained secondary or higher education did not change noticeably.

**Fig 4:**
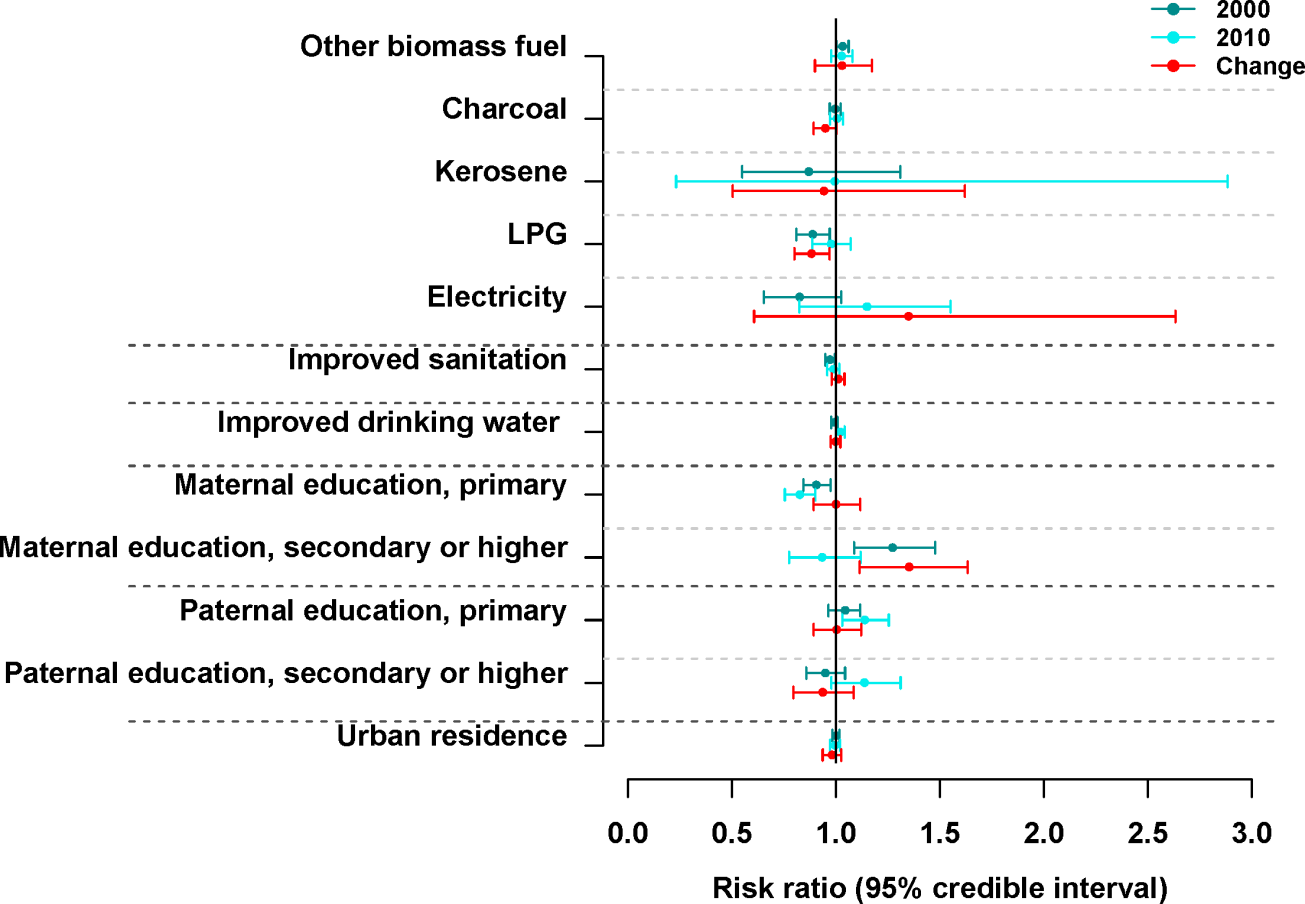
Risk ratios and 95% credible intervals (2.5th and 97.5th percentiles of the posterior distributions of effect size parameters from the Bayesian model) from multivariate analysis of the association of under-five mortality with its social and environmental risk factors for 2000, 2010, and the change between 2000 and 2010. The magnitude of the effect size for each variable represents the proportional change (decrease or increase) in 5q0 for a 10% higher prevalence of that variable (over space in a single census year or change over time between censuses), with the 10% shift coming from the reference variable. The reference variables were wood (for cooking fuel), unimproved sanitation, unimproved drinking water, no education (for maternal and paternal education), and living in rural areas.

In multivariate analysis, higher use of LPG for household cooking was associated with lower under-five mortality after adjusting for other factors, with each 10% increase in households using LPG associated with a 11.1% (95% CI 3.0%–18.8%) decline in 5q0 (Fig 4). Associations for the other social and environmental variables were not consistent or were weak in the different analyses, although there were indications of beneficial effects from replacing wood with charcoal or kerosene, from improved sanitation and drinking water, and from having a higher share of mothers who had completed primary education.

**Fig 5:**
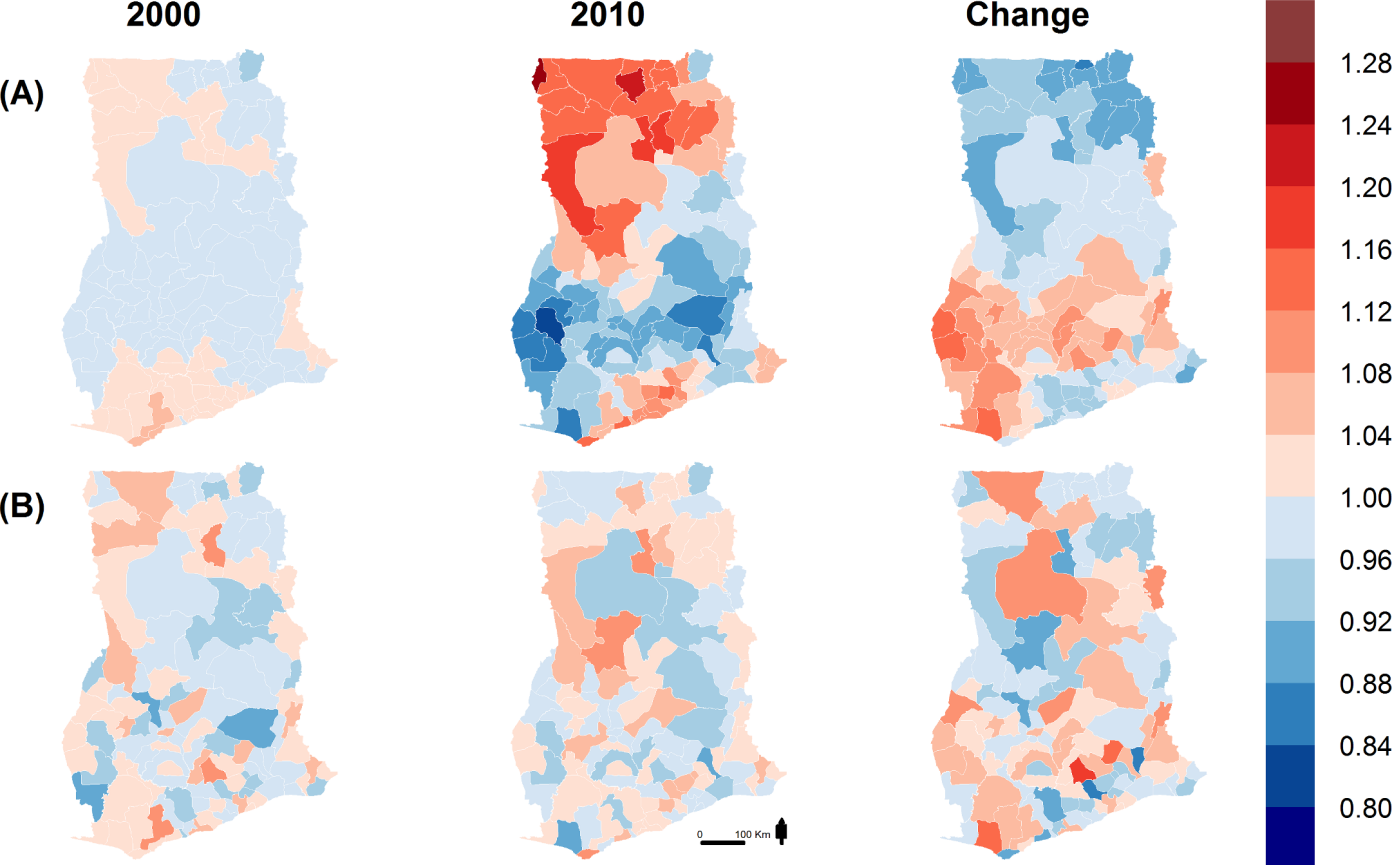
Unexplained district variability in under-five mortality. (A) Spatially structured random effects [exp(*U*)] and (B) unstructured random effects [exp(*V*)] of unexplained district variability in under-five mortality. Random effects < 1 for a district represents a reduction in under-five mortality, and random effects > 1 represents an increase.

After adjustment for socioeconomic and environmental factors, some unexplained spatial variation in under-five mortality remains (Fig 5), which represents unmeasured and/or unknown risk factors not already included in the model. Specifically, in 2010 the spatially structured part (Fig 5A) of the unexplained component of under-five mortality was higher in the northernmost and Greater Accra regions, and lower in the Western, Brong-Ahafo, Ashanti, and Eastern regions and parts of the Volta region (Fig 5A).

## Discussion

Our small-area analysis found that under-five mortality declined in all of Ghana’s districts between 2000 and 2010, but the size of this decline varied considerably across districts. The pace of decline was steeper in southern districts, where under-five mortality was lower in 2000, than in the north, exacerbating existing cross-district inequalities, especially for relative inequality. The decline in under-five mortality in Ghana appears to continue beyond 2010, as indicated by the latest national estimate by the UN IGME [2]. We also found improvements in education and household environment, including access to improved water and sanitation and cleaner cooking fuels, from 2000 to 2010. Higher use of LPG for cooking was associated with lower under-five mortality, but the associations of either the level or change in under-five mortality with other social and environmental variables were weak. We observed high unexplained variability in under-five mortality in parts of the country, especially in 2010. This unexplained variability does not readily point to any single factor that we are aware of. It is possible that a combination of unmeasured variables, including healthcare, socioeconomic status, natural resource availability, and local governance, played a role in this (unexplained) variability.

Like in our study, studies in Zambia, Papua New Guinea, and India found that under-five mortality declined in all or some districts over a similar time period, with large subnational variation in the magnitude of decline [9,14,19]. Unlike in our study, cross-district inequality decreased in Zambia over time, but the Indian study found a rise in cross-district inequality, as we did in Ghana. The association between parental, especially maternal, education and child survival was weaker in our data than in some other population-based studies [5,12,30–32].

The main strength of our study is its novel scope in analyzing under-five mortality, and its socioeconomic and environmental risk factors, at the small-area level. Small-area information allows benchmarking of subnational administrative units, both in terms of mortality levels and trends and in terms of the environmental and socioeconomic risk factors for mortality. This information can in turn identify districts that are performing well and those that are most in need of additional interventions related to the environmental and socioeconomic factors analyzed or to other determinants of child survival, as discussed below. We used geocoded data from two national censuses, which included every household in the country. We also used Bayesian spatial analysis, which balanced between unstable district-level estimates and simplified aggregate national estimates to obtain consistent and comparable under-five mortality estimates for all districts.

Our study also has a number of limitations. Due to the limited coverage of the vital registration system in Ghana, we relied on demographic models to estimate under-five mortality at the district level. While this approach is well tested and used by national and international agencies, it introduces uncertainty into our estimates. In particular, the estimates using the 2000 census were substantially different from the national estimates using other sources. We dealt with this issue by adjusting our estimates to be consistent with pooled estimates from all sources. This adjustment could introduce additional uncertainty into the district-level estimates. We could not separate child deaths into those during the neonatal versus subsequent periods. The social and environmental risk factors in our data were each measured using one or two questions in the census. This simplification of measurement may have affected the associations of these factors with under-five mortality. We had no information on healthcare access and interventions such as immunization, insecticide-treated nets, and nutritional supplementation, which are important factors for child survival [33–35]. Similarly, we had no information on within-country migration, which might be partially responsible for changes in specific districts over time.

### Conclusions

The Millennium Development Goals, and the policy emphases and resources that followed them, have led to acceleration of under-five mortality decline in Ghana and other countries in SSA. These efforts seem to be benefiting all of Ghana’s districts, but the rate of progress has been slow and unequal across districts. There is therefore a need both to accelerate the decline and to put special emphasis on districts that have progressed more slowly. Experiences of more equitable decline in countries such as Niger, Brazil, and Bangladesh show that achieving this requires multisectoral approaches while maintaining a major role for the health system [33–35]. In particular, although we found weak associations between under-five mortality and some of the social and environmental factors we investigated, the role of these factors may have been partially masked by others, including changes in health services and health systems interventions, and hence there should be investments in education and household environments as a way to continue and accelerate improvements in child survival. At the same time, the weak association of child mortality with social and environmental factors, and the large body of evidence on the efficacy of healthcare and health systems interventions for child health, should motivate increasing the coverage of healthcare interventions such as antenatal care, immunization, insecticide-treated nets, and treatment of acute conditions such as pneumonia, malaria, and diarrhea throughout Ghana, with an emphasis on equity in access and utilization [33,36]. Finally, improving measurement and monitoring of under-five mortality and its risk factors and interventions at the subnational level are particularly important for all SSA countries, to better inform policies and programs that facilitate equitable progress.

## Supporting Information

S1 Data Counts of children ever born, children surviving, and women of childbearing age by age group, district, and census year. (XLSX)

S2 Data Socioeconomic and environmental variables by district and census year. (XLSX)

## Abbreviations

EA: enumeration area
IGME: Inter-agency Group for Child Mortality Estimation
LPG: liquefied petroleum gas
SSA: sub-Saharan Africa
